# Local Perceptions, Cultural Beliefs and Practices That Shape Umbilical Cord Care: A Qualitative Study in Southern Province, Zambia

**DOI:** 10.1371/journal.pone.0079191

**Published:** 2013-11-07

**Authors:** Julie M. Herlihy, Affan Shaikh, Arthur Mazimba, Natalie Gagne, Caroline Grogan, Chipo Mpamba, Bernadine Sooli, Grace Simamvwa, Catherine Mabeta, Peggy Shankoti, Lisa Messersmith, Katherine Semrau, Davidson H. Hamer

**Affiliations:** 1 Center for Global Health and Development, Boston University, Boston, Massachusetts, United States of America; 2 Department of Pediatrics, Boston Medical Center, Boston, Massachusetts, United States of America; 3 Department of International Health, Boston University School of Public Health, Boston, Massachusetts, United States of America; 4 Zambia Center for Applied Health Research and Development, Lusaka, Zambia; 5 Agha Khan Development Network, Mopti, Mali; 6 Section of Infectious Diseases, Department of Medicine, Boston Medical Center, Boston, Massachusetts, United States of America; Edinburgh University, United Kingdom

## Abstract

**Background:**

Global policy regarding optimal umbilical cord care to prevent neonatal illness is an active discussion among researchers and policy makers. In preparation for a large cluster-randomized control trial to measure the impact of 4% chlorhexidine as an umbilical wash versus dry cord care on neonatal mortality in Southern Province, Zambia, we performed a qualitative study to determine local perceptions of cord health and illness and the cultural belief system that shapes umbilical cord care knowledge, attitudes, and practices.

**Methods and Findings:**

This study consisted of 36 focus group discussions with breastfeeding mothers, grandmothers, and traditional birth attendants, and 42 in-depth interviews with key community informants. Semi-structured field guides were used to lead discussions and interviews at urban and rural sites. A wide variation in knowledge, beliefs, and practices surrounding cord care was discovered. For home deliveries, cords were cut with non-sterile razor blades or local grass. Cord applications included drying agents (e.g., charcoal, baby powder, dust), lubricating agents (e.g., Vaseline, cooking oil, used motor oil) and agents intended for medicinal/protective purposes (e.g., breast milk, cow dung, chicken feces). Concerns regarding the length of time until cord detachment were universally expressed. Blood clots in the umbilical cord, *bulongo-longo*, were perceived to foreshadow neonatal illness. Management of *bulongo-longo* or infected umbilical cords included multiple traditional remedies and treatment at government health centers.

**Conclusion:**

Umbilical cord care practices and beliefs were diverse. Dry cord care, as recommended by the World Health Organization at the time of the study, is not widely practiced in Southern Province, Zambia. A cultural health systems model that depicts all stakeholders is proposed as an approach for policy makers and program implementers to work synergistically with existing cultural beliefs and practices in order to maximize effectiveness of evidence-based interventions.

## Introduction

Despite considerable reductions in the under-five mortality rate in Zambia since 1990, infant and neonatal mortality rates remain undesirably high and preventable[1]. According to the 2007 Zambia Demographic and Health Survey (DHS), the under-five mortality rate was 119 deaths per 1,000 live births, a disproportionate number of these deaths occur in the first four weeks of life as evidenced by a neonatal mortality rate of 34 deaths per 1,000 live births [2]. A United Nations Statistics Division 2012 progress report indicates that the under-five mortality rate has decreased to 82.9 deaths per 1,000 live births; at this pace, it is unlikely that Zambia will meet its Millennium Development Goal target of 61 deaths per 1,000 live births by 2015 [3]. There is a global push from policy makers and public health practitioners to implement effective evidence-based interventions to reduce newborn deaths of which the leading causes are infection, birth asphyxia, complications due to prematurity, and congenital abnormalities [4-6]. Umbilical cord infections (omphalitis) and neonatal sepsis are significant contributors to the proportion of neonatal infections that prove fatal. Research from Nepal, Bangladesh and Pakistan demonstrates the efficacy of 4% chlorhexidine when used as an umbilical wash to lower omphalitis risk and neonatal mortality [7-10]. This evidence has led global policy makers to focus on creating umbilical cord care policy and practice guidelines. In order to create effective health policy and programs, qualitative research is needed to better understand the cultural context of umbilical cord care. It is essential to identify how key stakeholders perceive, understand and react to newborn cord health and illness. Several studies have already described various aspects of newborn care worldwide[9,11-24].

Most of what we know about cord care practices comes from Southeast Asian cultures; there is little information about cord care practices in sub-Saharan African and almost none from Zambia. A sole qualitative study published in 2003 explored cultural childbirth practices in Zambia, but did not discuss cord care [25]. Maimbolwa et al conducted 36 in-depth interviews (IDIs) with birthing companions in two urban and eight rural Zambian health facilities to assess common traditional practices and care seeking surrounding labour, delivery and post-natal care of mother and newborn. The authors highlighted the role that support women play during labor; however, no mothers were interviewed and no home birth experiences were recorded. In Zambia, more than half of women deliver at home for several reasons including lack of transportation, lack of access, lack of funds, and by preference [2,26]. Home deliveries in low resource settings and births attended by unskilled birth attendants have long been implicated in increasing the risk of neonatal tetanus, omphalitis, and sepsis [27-32]. Understanding what happens to newborns and their umbilical cords during a home birth and a facility birth are vital to planning interventions that may require behavior change.

Newborn care varies widely across cultures; the forces and persons that shape newborn care, perceptions of illness, disease and thresholds for care seeking are unique to each context. The success of public health interventions and uptake of policies are dependent on understanding the cultural context that shapes the practices, underlying beliefs and collective constructed meaning for cord function, cord health and disease. This paper shares the findings of a formative qualitative study that was performed in Southern Province, Zambia to inform the design and procedures of a large cluster-randomized trial to determine the impact of 4% chlorhexidine versus dry cord care on neonatal mortality. The objective of this qualitative study was to investigate the practices, beliefs, attitudes and perceptions of umbilical cord care and illness in newborns in Southern Province of Zambia. Our goal was to use these findings to shape our approach to the quantitative study and to inform future policy or programming related to cord care in Zambia. 

## Methods

**Figure 1:**
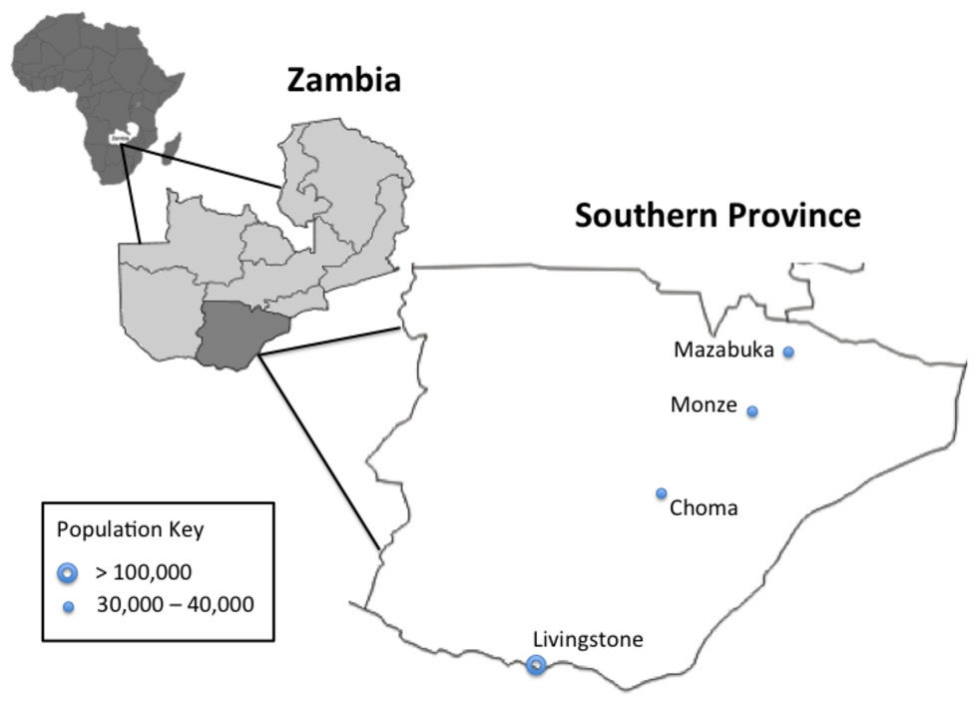
Map of Southern Province, Zambia.

We conducted 36 focus group discussions (FGDs) comprised of 339 respondents and 42 in-depth interviews (IDIs) in Southern Zambia. Data collection occurred in two rural locations (Choma and Monze Districts) and two urban locations (Livingstone and Mazabuka Districts) [Figure 1] between February and April 2010. Settings were purposively chosen to be located away from health facilities and without the presence of known authorities (health facility staff or tribal leadership) to encourage openness and reduce reporting bias regarding care seeking behaviors. The setting in Choma District was a community meeting house near the chief’s palace. In Monze and Mazabuka Districts, an empty schoolroom was used for focus groups and interviews. In Livingstone, all IDIs and FGDs took place in a community meeting room near the health facility. In all districts, health facility staff members were interviewed at their health facility in order to minimize interruptions to patient care. For all FGDs and IDIs, only the participants and interviewers were present. 

To recruit and identify participants, interviewers met with health facility staff, neighborhood health committee members and village headman to identify traditional birth attendants. Of note, Republic of Zambia Government formerly had a national training program for traditional birth attendants. This program ceased in 2010, but many of its graduates still actively attend deliveries in the community and some work at health facilities (they are locally referred to as “trained” TBAs). A snowball method that started with the trained TBAs as the primary point of contact was then used to identify and invite breastfeeding mothers, grandmothers and other TBAs who were not formerly trained but actively attend deliveries (these women are locally referred to as “untrained TBAs”). Thirteen FGDs were conducted with breastfeeding mothers, 12 FGDs with grandmothers of children under-5 years of age, 10 FGDs with trained and untrained TBAs. One FGD was conducted in Livingstone with health facility midwives. FGDs with each of the above designated groups were performed in each district in order to capture perspectives from mothers, grandmothers and TBAs from urban and rural settings. IDIs were performed with key informants in the community, including community and religious leaders, community health workers (CHWs), health professionals, midwives, traditional healers, and trained and untrained TBAs. 

Interviewers were all retired midwives who previously worked for the Government of Zambia’s health sector. Interviewers underwent training in ethics and qualitative interviewing. All IDIs and FGDs utilized a semi-structured guide, which asked initial questions and provided probe questions if needed. Interview guides were pilot tested with volunteer mothers and grandmothers. Pilot sessions were audio-recorded to allow for reflection and feedback from the researcher on interview style. Interview guides were adapted post-piloting and submitted to the respective institutional review boards and ethics boards prior to use. 

Most FGDs were conducted in Tonga, the predominant local language in Southern Province. Two FGDs were supplemented with Nyanja, another local dialect. This was done in Livingstone, a border town, which has more linguistic diversity. Lastly, one FGD with midwives was conducted in English. All FGDs and IDIs were audio-recorded and later translated into English and dictated and transcribed by Dr. Herlihy and two research assistants who are primary English speakers. Transcripts were made anonymous and uploaded into NVivo software version 9.0 (QRS International, Cambridge, MA). Transcripts were numbered and sorted randomly; four independent reviewers coded 50% of the transcripts each, allowing for double coding of each transcript. Key themes were identified by each coder and discrepancies were reconciled during group memoing and review. Using a deductive approach, known themes of cord application and cord cutting procedures were explored and emerging themes were identified using thematic analysis. Models of care seeking, belief constructs, and practices evolved from the themes generated and are shared in the discussion. COREQ reporting guidelines were followed in the study design and in preparation of the manuscript. 

### Ethics Statement

The protocol and consent forms were reviewed and approved by the Boston University Institutional Review Board (FWA# 00008404) and the University of Zambia Research Ethics Committee (FWA# 00001131). The consent forms were translated into Tonga and English; a certified bilingual speaker attested to the accuracy of the translation. Written informed consent was obtained from each participant. All participants were read a translated Tonga version of the consent form in a group (for FGDs) or individually (for IDIs). Participants were given an opportunity to opt-out of participating or to ask clarifying questions. Each participant provided documentation of consent with a signature, mark, or thumbprint; copies of the consent forms were provided to participants.

## Findings

### Study Population Characteristics

A total of 339 eligible individuals participated in 36 FGDs, with seven to eleven individuals per group. Nine FGDs were conducted in each rural (Choma and Monze) and urban (Livingstone and Mazabuka) site. Demographics of participants are presented in Table 1. 

**Table 1:** Study Participant Demographics.

Focus Group Discussion (FGD)	Number of FGDs: n= 36	Number of participants n=339	% Rural	% Female
Grandmothers	12	117	50	100
Breastfeeding mothers	13	118	53	100
Midwives	1	8	0	100
Mixed trained and untrained TBAs	4	39	75	100
Untrained TBAs	6	57	33	100
In-Depth Interviews (IDI)	Number of IDI: n= 42	Mean age (range)	% Rural	% Female
Community Health Workers	2	35*	100	50
Community Leaders	8	49.8 (43-53)	62.5	25
District Level Health Professionals	7	53.4 (49-61)	28.5	42.8
Midwife	6	42.8 (33-59)	50	83.3
Religious Leader	4	51.5 (40-65)	50	0
Trained TBA	2	57.5 (57-58)	50	100
Untrained TBA	5	49.6 (39-58)	60	100
Traditional Healer	8	54.75 (47-68)	50	50

*Age for one Community Health Worker was not available

Of the 42 IDIs, 11 took place in each rural site (Choma and Monze) and 10 took place in each urban site (Livingstone and Mazabuka), with a total of 22 (52.4%) females participating [Table 1]. Age of respondents ranged from 33 to 68 years old, with a median of 51 years. In total, eleven tribes were represented, with the majority belonging to the Tonga tribe (n= 26, 62%), followed by the Lozi, Kaonde, Ngoni, Ila, Kalunda, Lala, Mambwe, Nkoya, and Nyika with five or less representatives each. On average female participants had approximately five children each (range 1-10 children); male respondents had a mean of seven children each (range 3-13 children).

### Cord Function

Most respondents described the purpose of the umbilical cord as a means of providing nutrients to the growing fetus.

“The cord is where the baby feeds from”.

However, a number of responses demonstrated alternative syncretic beliefs, as demonstrated in the following statement explaining the role of the umbilical cord:

“It [the umbilical cord] has a function. That's where the baby's heart is. Because the cord is connected to the baby, that's why I say the cord is the heart of the baby, especially when the baby is in the womb.” (IDI 30)

-54 year old, female traditional healer, Livingstone 

A number of associations were made between the actions of the mother or father and the well being of the baby. Examples of actions that are associated with poor infant health include mothers putting salt in their food or fathers being engaged in extra-marital relations. Also, many respondents spoke of a belief that wearing certain clothes (e.g. father wearing a neckties, mother wearing necklaces) influences the length of the umbilical cord, as illustrated by the comments below:

“If one wants to put things around the neck when you are pregnant, then even the cord will be long, then it will go around the baby's neck because of you putting things around your neck.” (IDI 4)

-35 year old, male CHW, Choma

“Mothers are advised not to tie belt, carry hand bags or tie *chitenge* (fabric wrap) very tight because the baby will be born with cord around the neck and arm and the baby can die” (IDI 8)

-50 year old, female untrained TBA, rural Choma

### Delivery Preparation

Universally, respondents reported that mothers are primarily responsible for all delivery-related preparations. In Zambia, mothers are expected to purchase commercially available materials and tools needed for a clean delivery (e.g. gloves, clean razor, cotton cord tie or clamp) regardless of desired location of delivery (home or facility). For home deliveries, mothers also needed to identify and contact a TBA to perform the delivery at home and to create a clean area in the home for delivery. Timing of preparation varied, but commonly respondents noted that mothers begin preparing for delivery around the 7^th^ month of pregnancy. TBAs reported instances when they provided tools to the pregnant woman at the time of delivery if the delivery was precipitous. There was a degree of shame expressed regarding women who had not prepared the materials necessary for delivery.

“Grandmother: Every pregnant woman should prepare for her delivery. She should buy a blade and a cotton; if she doesn’t have [money] she should prepare the *dezhya* [grass used to cut the cord]. If using the *dezhya*, she should clean it. If not, the blade is clean from the shop. The dezhya is also clean because it’s from the bush and that prevents infection of the cord.

Facilitator: Who is supposed to prepare all these things? Make sure they are clean?

Grandmother: The mother, the delivering woman should keep all these things. So at the time of delivery the mother will just bring everything clean.”

### Cord Tying, Cutting, Timing, and Length

The umbilical cord was most frequently tied with white or black cotton knitting wool during home deliveries, and with cord clamps if delivered at a health facility. In emergencies, women may use a part of their *chitenge* (traditional fabric wrap) to tie the cord; however, this was perceived as harmful to the baby due to risk of infection and reflected poorly on a mother who was considered “unprepared” for delivery.

“Sometimes you will come across the baby [who is] born at home and is just tied with some dirty thread or pieces of cloth to tie the cord.” (IDI 2)

-56 year old, male religious leader, Choma 

Focus group participants described an older practice that is no longer performed of tying the cord with *loozi*, a fiber from the bark of a tree. Three knots are tied into the cord, one knot destined to remain with the mother and two to the baby’s side of the cut cord. 

Respondents said the person conducting the delivery, whether it is a nurse, midwife, TBA or family member would also be the one to cut the cord. A number of respondents both urban and rural from the mothers, grandmothers and TBA focus groups reported a cultural tradition linked to cutting the cord. It is a call and response style chant that compares the umbilical cord to an elephant, which takes place while cutting the cord.

“The one who is delivering […] cut[s] the cord while singing: *Mutendanzi*? (What are you cutting?) The one who is asking is the TBA. The response is *Tu tenda* *muzovu* (We are cutting an elephant), and the ones who are answering are the other people who are present during the delivery. People used to rejoice when somebody has delivered. Because the one who was pregnant was tied up, now she is free.” (IDI 16)

-52 year old, female traditional healer, Mazabuka

When asked to explain the significance of this practice, participants stated that calling the umbilical cord ‘the elephant’ may represent multiple things including: the burden of being pregnant is as large as an elephant, and the woman is now free; the wish for the baby to be an important member in society as the elephant is an important and respected animal in the savannah. Many FGD participants did not know a deeper meaning of this chant, but had participated in it because it was a symbolic custom handed down for generations that had ‘lost it’s meaning.’ 

A number of tools are used to cut the umbilical cord— separating the baby from the placenta; nearly all respondents referred to the razor blade as the tool of choice due to its affordability, efficiency, and capacity for single-use. This is especially important in the context of the HIV epidemic as indicated by the following statement:

“These days with HIV, they are taught to prepare all things they need including new blades.” (IDI 1)

-51 year old, male community leader, Choma

The most frequently mentioned alternative tool to razor blades are scissors, which were typically used in a clinic setting. A few participants pointed to the use of other tools that were considered traditional and no longer used, including a variety of organic materials such as: grasses, sugarcane, reed and maize stalk peelings (referred to locally as *dezhya* in Tonga), and sharpened stones or metals (referred to locally as *lunyoolo* in Tonga). Although these tools were not favored, there was recognition that in emergency situations the use of traditional tools was an appropriate alternative approach for cord cutting.

“I went to conduct a delivery, they gave me an old blade to use, I refused, then they brought an old knife from the kitchen, I said ‘I can’t use a knife as if I am cutting meat’ […] I went and got a reed from a mat, cleaned it nicely and used it to cut the cord.” (IDI 29)

-45 year old, female untrained TBA, Livingstone

Nearly all of the traditional healers and the majority of TBAs reported they did not sterilize tools prior to use. However most of the CHWs and midwives reported some sort of sterilization process. Sterilization techniques mentioned included: boiling tools for 20-30 minutes or sterilizing with spirit (ethanol) only if they were intended for repeat use. The prevailing sense was that if something is new it is clean and ready to be used immediately. Participants reported that tools such as grasses and sugarcane or maize stalk peelings used to cut the cord are never boiled due to a loss in physical integrity. Instead organic tools are considered naturally clean and therefore are minimally sanitized as depicted in the following statements.

“[The grass] it’s supposed to be clean, just as God made it.” (IDI 5)

-56 year old, female untrained TBA, Choma

The timing of cord cutting with respect to placental delivery varied widely. Reasons provided for cutting the cord prior to placental delivery mostly related to safe delivery of the infant. One respondent commented:

“We cut the cord before the placenta is delivered because sometimes a baby can be delivered with the cord around the neck or the body, so if you don’t cut the cord it can strangle the baby and the baby can die.” (IDI 16)

- 52 year old, female traditional healer, Mazabuka

Conversely, a number of respondents favor delaying cord cutting until after the placenta is delivered due to concerns for a retained placenta or products of conception. One respondent emphasized the importance of timing, stating:

“It is even taboo to cut the cord before the placenta comes out, the placenta has roots in the mother and it will be difficult for it to be removed […] Those roots just stick to the uterus so that can even kill the woman because it will not come out easily, even if they try by all means to squeeze the abdomen of the woman.” (IDI 28)

- 68 year old, male traditional healer, Monze

The importance of cord length is reported by a number of participants. To estimate where the cord ought to be cut, birthing attendants may use any adult finger excluding the thumb, the baby’s thigh or knee or in a few cases the baby’s own finger to estimate the length, which is on average 3-5 cm long. There was some conflict over the perceived appropriate cord length as illustrated by the statements below. Some participants expressed concern that healing would take too long if the cord was too long; this may prompt cord applications to accelerate the rate of drying. Others expressed concern that a cord cut too short that would foreshadow short genitalia or cause harm to the newborn.

“Yes, it [cord length] matters. Because the baby breathes from the cord as others have said; if the cord is too short, the air will go in and this will make the baby die. If it is too long, again, it will take time to heal.” (FGD 35)

- Age unknown, grandmother, Livingstone FGD participant

“[Under the Lozi tradition] if it’s a boy, and if you cut the cord long, than the penis will be long […] If it’s a girl, we tie the cord in three places. The reason why we do this because we want when she will be grown up then when she will start preparing herself to be a woman, her labias will be long and her back will be strong.” (FGD 18)

- Age unknown, untrained TBA, Mazabuka FGD participant

Following cutting the cord that separates the baby from the mother, a few respondents reported the cultural practice of applying the bloody end of the cut cord to both the mother and the newborn. In both reports of this practice, the cut cord is pressed to the forehead of the mother. However there was no reported meaning given for this action.

“We cut and tie the cord, then we make a dot (of blood) on the mother’s and baby’s forehead using the end of the cord.” (FGD 24)

- Age unknown grandmother, Monze FGD participant

### Cord Applications

**Figure 2:**
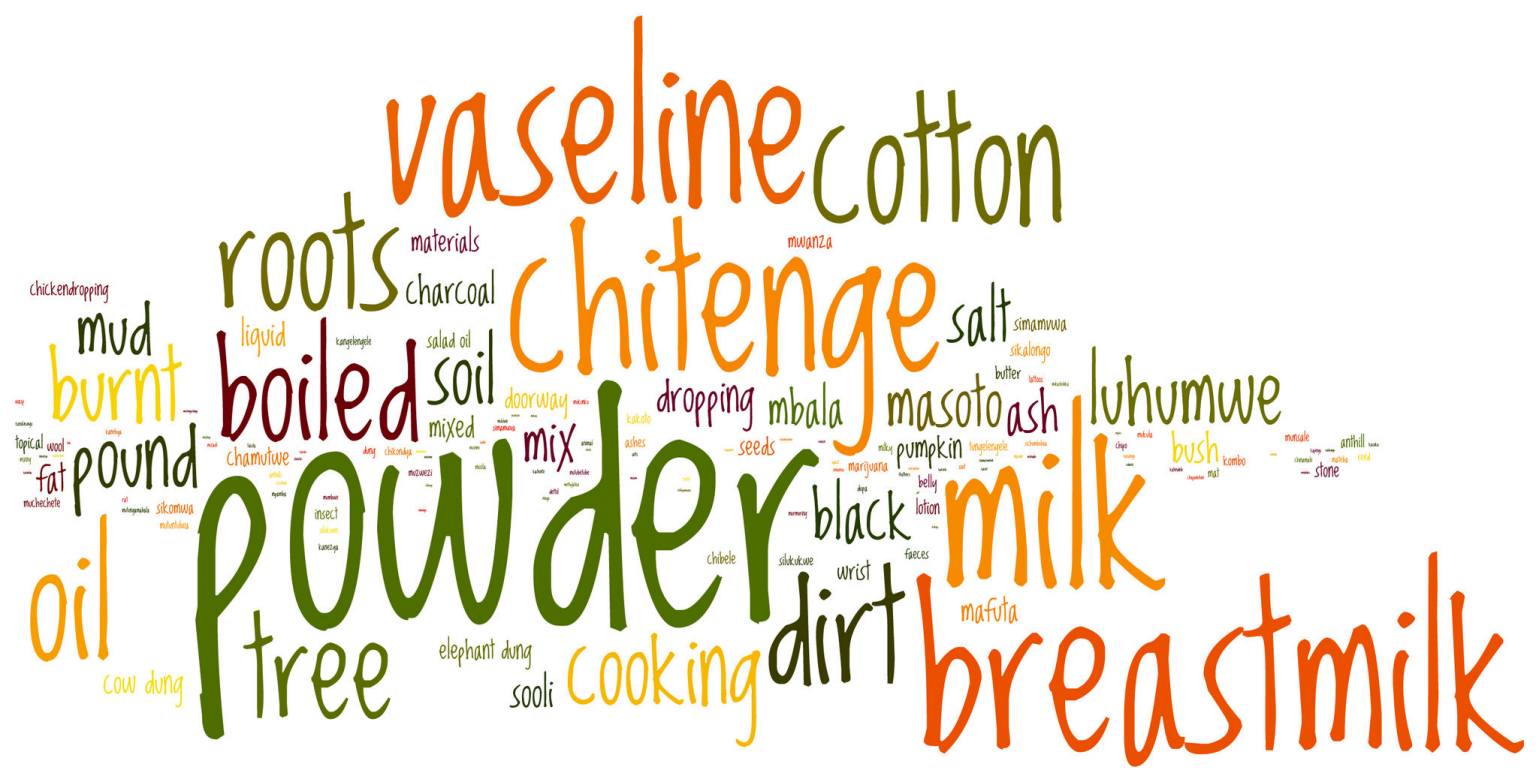
Word Cloud of Cord Applications. The size of the font represents the frequency at which this term was mentioned in focus group discussions and in-depth interviews.

A myriad of substances were reported as cord applications. Figure 2 is a word cloud of all mentioned cord applications; the size of the word corresponds to the frequency with which the word was mentioned in the transcripts. Rationales for applying different types of substances varied from lubricating agents to drying agents and lastly, medicinal agents if infection was suspected. If a cord stump is viewed as being too brittle, is cracking or bleeding, a substance that would increase moisture or “softness” of the cord may be considered. Examples include breast milk, Vaseline®, cooking and motor oil, *mabono* (wild fruit) oil, or cream from sour milk. Vaseline® was one of the most common items applied and was often recommended by TBAs or grandmothers. Warmed cooking or used motor oil were uncommon practices, but were sometimes used as a base for other ingredients (e.g. charcoal, herbs) to create an oil-based balm. Another untrained TBA explained the preparation for *mabono* oil, a commonly found fruit that had many reported uses [Table 2]. 

**Table 2:** Comments Regarding Cord Applications.

Theme	Comments	Respondent Characteristics
Cord applications	“You crush the charcoal, mix it with cooking oil, and apply to the cord. The reason why I put charcoal and salad (cooking) oil is for the cord to remain soft. It will not hurt the baby.”(FGD 35)	Age unknown, Grandmother, Livingstone
	“They used mabono (a fruit from a local tree)…they used to fry them, then pound them and then put them in boiling water, then take the oil that comes on top and it was scooped out with a spoon or bark of a tree and used to apply” (IDI 5)	56 year old, female untrained TBA, Choma
Substances to accelerate cord detachment	“Others used to burn the stalk of the pumpkin…others used chicken droppings mixed with cooking oil, then you take a chicken feather and use it for applying on the cord at the root…so that it dries off quickly.” (FGD 15)	Age unknown, TBA, Mazabuka
	“After the cord drops, you take cockroaches, burn them and crush into a powder and apply on the baby’s cord until it heals.” (FGD 32)	Age unknown, untrained TBA, Livingstone
	“I also saw people pounding tusumbwa (black small anthill in swampy areas) then take ‘bulongo’ (dirt from anthills) and put it on the cord for fast drying” (IDI 4)	35 year old, male CHW, Choma
	“If you want the cord to separate quickly, you will get ‘muuye’ (the nest of a biting wasp)…[pound into] a powder, you just put on the umbilical cord.” (IDI 7)	39 year old, female untrained TBA, Choma
	“The mother puts breast milk, sometimes they use dirt from the main door entrance so that the cord can dry and drop fast.” (IDI 8)	50 year old, female untrained TBA, Choma
Medicinal applications	“There is something they put and it’s called mafuta mbooma (oil from the Python snake).” (IDI 34)	54 year old, male traditional healer, Livingstone
	“Like me, if there is pus discharge on the cord, that’s when I put charcoal powder so that it can heal quickly.” (IDI 30)	54 year old, female traditional healer, Livingstone

When a cord is deemed to be taking longer than expected to fall off, a drying agent might be used to accelerate the detachment process. Substances reported include baby powder, charcoal dust, dried cow dung, dried chicken droppings, dust from the threshold of the home, ash from a burnt pumpkin handle, crushed *loma* (wasp nest) or mud. Each of these items is ground or pounded into a fine powder prior to application [Table 2]. Additionally, many of the substances described were also used as medicinal agents or used solely for medicinal purposes. Medical substances include breast milk, alcohol, python snake oil, banana, cow dung, *mukunku* (bark of a tree), traditional herbs, and the dirt from pounding stick. Indications for use would be redness of the cord or the appearance of pus or vesicles.

“if the child was born at the hospital, the cord falls off, you’ll find that where the cord was, there is a reddish sore inside […] even if you apply powder to treat it, it can go up to 2 months, it will not heal.  You will find tomorrow that it is very red [so] you get the roots from the bananas, stool from a goat, you burn them, and mix.  You also mix it with oil, then you start applying on the cord.  So it only takes two days, that sore will heal.” (FGD 10)

- Age unknown grandmother, Mazabuka, FGD participant 

Breast milk had multiple indications for cord applications. Some reported its use to prevent infection; others to treat an infection that is present; and lastly, some reported that it is used as a lubricating agent to soften the cord. Timing of cord application differed depending on the rationale for the use. Lubricating agents (e.g. Vaseline®, breast milk, oil-based agents) and those intended to protect against infection (e.g. breast milk) appeared to be applied soon after birth, whereas some drying agents (e.g. charcoal, dust, dung, powder) were applied to accelerate cord dropping, if the cord was not falling off as expected (5-7 days of life). 

Not everyone reported applying substances to the cord, but there was little familiarity with the concept that dry cord care, or allowing the cord to dry on its own without any applications, is the Zambian Ministry of Health and World Health Organization policy [33,34]. Health workers reported a variety of witnessed cord applications but expressed little concern over some types of applications (e.g. baby powder, Vaseline® or breast milk). Although no health workers from the government clinics reported recommending these applications, only one of them reported counseling against it. There seemed to be little knowledge of dry cord care as an active clinical guideline. One conversation between an interviewer and a 56 year old neighborhood health committee member illustrates the conflict between practice and understanding of recommendations.

“Neighborhood Health Committee Member (NHCM): There are things [cord applications] that are recommended to be used on the baby; things like car oil are not recommended.

Interviewer: What do they do to the baby?

NHCM: They bring sickness to the baby.

Interviewer: Now tell about what is applied on the cord, you have eight children, what have been using on your children?

NHCM: I have been using lotion, but for those that didn’t have baby lotion they used oil.

Interviewer: What type of oil?

NHCM: Car oil.

### Perceptions of Disease and Care Seeking Behavior

**Figure 3:**
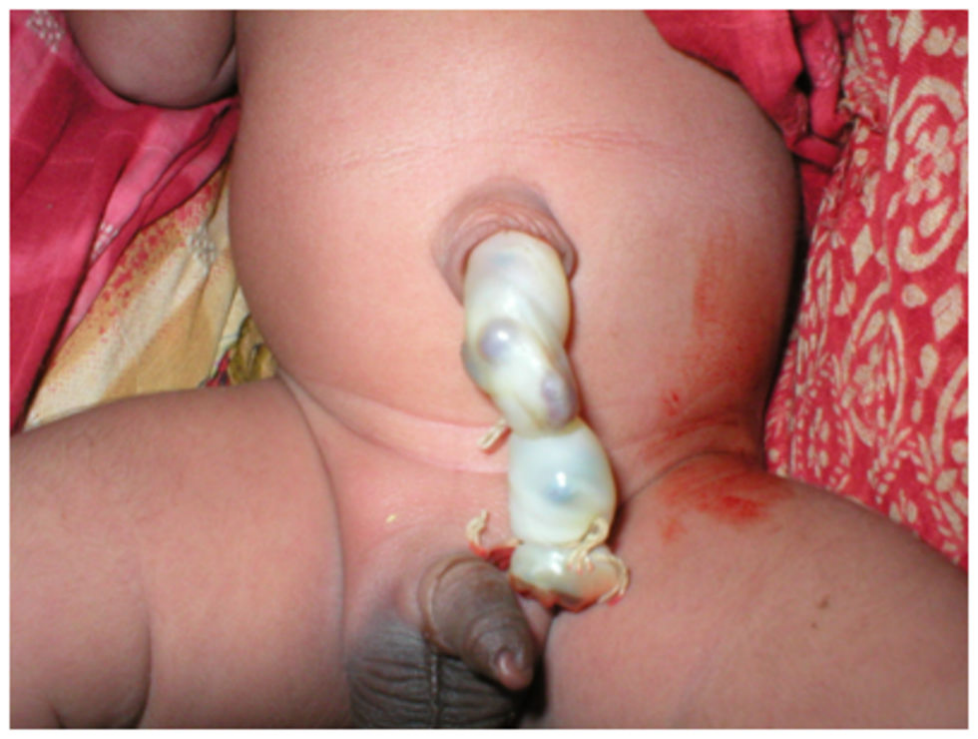
*Bulongo-longo*. Blood clots in umbilical vein are called *bulongo-longo* in Chitonga and thought to foreshadow ill health. (Photo Credit: Luke Mullany PhD, Tanzania).

Respondents reported cultural practices associated with the presence of *bulongo-longo* (black bumps in the cord). The black bumps referred to are benign blood clots in the umbilical vein [See Figure 3]. Respondents associated the presence of *bulongo-longo* as the newborn being vulnerable to a curse, which may then lead to an increased risk for catching *masoto* (respiratory illness) and *luhumwe* (distended abdomen). On observation of *bulongo-longo*, caregivers reported taking measures to reduce risk of their child being cursed and facing subsequent disease. These included manual extraction of blood clots, consumption of a preventative broth made from anthill soil, wasp nests, and dung, or indoor confinement ranging from 1-3 months. It appeared that there was a window of time (although not defined) in which a curse could be averted if preventative measures were taken quickly after birth. A description of *bulongo-longo* and one approach to reducing its effects is included below [Figure 3].

“It looks like goat droppings. When the baby is born, you have to pinch *bulongo-longo* back toward the mother, then tie and cut […] in doing so, the baby only needs to stay in the house for a week, not one month.” (FGD 8)

- Age unknown grandmother, Choma FGD participant

Other disease states concerning the umbilical cord described by participants included a cord with redness, discharge or pus.  The majority of respondents stated that this condition likely represented an infection and should be taken to a medical clinic for evaluation.  Alternative approaches included seeking care with a traditional healer; applying “spirit”  (ethanol) to the cord to see if redness resolved; removing the pus with a cloth used for diapers or a chicken feather; washing with soap or salt water; or applying breast milk, Vaseline®, ground *loma* (wasp nest), or ground powder from a burnt brick.  There was some conflict over the purposes of these applications; some participants identified these same applications as causing the infection as opposed to having medicinal properties to heal the infection.  Other causes of infection included a cord that was tied improperly, “cut wrong,” or a baby who was not carried on the mother’s back, thus, not allowing the cord to dry and drop properly. Mothers and grandmothers often described seeking care from multiple providers ranging from clinic staff to elders to traditional healers.  

### Cord Stump Detachment

When asked how long the cord usually remains attached, many participants remarked that the length of time should equal the same number of days as the mothers’ menses. Any longer may prompt a cord application to accelerate the process. Responses for the acceptable time for cord detachment ranged from 2 to 7 days. 

Respondents believed that a shorter time for cord detachment was better. The baby was considered vulnerable to disease exposure until the cord fell off, and the mother was said to experience associated abdominal pains called *chifufuti* in Tonga. A cord remaining for longer than 7 days was considered taboo and at times making people fear the child for being unusual.

Many respondents reported a number of precautionary measures mothers should take prior to cord detachment to safeguard the child, including confining the mother inside the home as well as restricting her movements, actions, and household chores until the cord fell. This was reported often but was not a universally accepted among respondents. Respondents felt strongly that the newborn should not leave the home until the cord falls off. Furthermore, visits to the home by pregnant or menstruating women, as well as a husband’s infidelity are perceived as detrimental to the child’s health and possible causes of prolonged cord detachment [Table 3].

**Table 3:** Comments Regarding Cord Detachment.

Theme	Quotes	Respondent Characteristics
Duration of attachment	“The time it takes, means it’s the time the lady takes to have her period.” (IDI 8)	50 year old, female untrained TBA, Choma
	“It depends on [the] tribe, but […] it takes the length of time you take during your menses.” (FGD 14)	Age unknown, breastfeeding mother, Mazabuka
Longer durations	“If it doesn’t drop fast, then there will be dirty air going through the umbilical [cord] and this will cause problem inside the baby. It's better that it drops off quickly so that it closes.” (FGD 24)	Age unknown, grandmother, Monze FGD participant
	“Shorter time is good, three days; the reason why I have said this is the longer the cord takes to separate, the mother will have more pains.” (FGD 27)	Age unknown, breastfeeding mother, Monze
	“If the cord takes more than 5 days and goes up to 7 days or more to fall, at that stage the baby starts even to give you a smile, this baby is growing and this baby starts to recognize people. This is taboo to the village. This cord needs to drop while the baby is very very small […] The village will be frightened if this happens.” (FGD 19)	Age unknown, TBA, Monze FGD participant
Precautionary measures	“The mother has to remain in the house until the cord drops. They say if the mother comes out, then she will start cooking and doing other things, so she's not allowed. Before the cord falls off, the mother is not supposed to handle anything in the kitchen. Or even put salt in food. That’s why they are kept separately in the bedroom […] It is believed if she puts salt in the food and some males eat from that food they will have a terrible chronic cough.” (FGD 36)	Age unknown, Midwife TBA, Livingstone FGD participant
	“The baby is not supposed to be seen by people until the cord drops.” (FGD 14)	Age unknown, breastfeeding mother, Mazabuka FGD participant
	“If a pregnant woman comes to see this baby, the baby will have a cracked fontanel, so this baby should remain in the house so that people don't go in to see the baby.” (IDI 34)	55 year old, male traditional healer, Livingstone
	“Those people who come to see the baby are the ones who cause the cord to delay to drop. If also the father has got another woman somewhere, than it will bring ‘masoto’ (bad air or spirits) and it will also delay the cord to drop.” (IDI 17)	39 year old, male traditional healer, Mazabuka

When the cord finally does drop, a large number of respondents reported associated cultural beliefs and practices. Chief among them, it is considered taboo and an ill omen for the cord to drop in the pubic or groin area. Failure to prevent this was perceived as detrimental to the baby, resulting in future infertility and an additional loss of sexual appeal in females.

“If it's a woman, she will be cold […] she won't be a proper woman, because the cord is cold. That means herself also she will be as cold as the cord in the private part, she won’t be a real woman.” (IDI 20)

- 50 year old, male health professional, Monze

Respondents reported advising mothers on several ways to safeguard their newborn once the cord has dropped. To protect the cord stump from falling on the pubic area respondents recommended vigilance and precautionary measures including tying a piece of cloth around the baby’s abdomen.

“the cord will be observed carefully so that it doesn’t drop on the pubis, because if it does that child will be sterile, whether male or female, so you have to put a piece of cloth to cover the lower part of the baby's abdomen.” (IDI 29)

- 45 year old, female untrained TBA, Livingstone

Once the cord has fallen, respondents recommend giving the baby to a young child to take out into the village to prove the baby’s strength; this practice was seen as protective to the newborn to shield her or him from future disease and aid the stump in healing faster.

“After the cord has dropped off […] in the morning, the woman who has delivered will [go] out to the river and take a cold bath and when she comes back from the river, that’s when now they will get a small child from the village, and give that child the baby, and will ask that child to go out with the baby in the village and then come back to the house later. That prevents the baby from getting diseases. After the child comes back with the baby you are assured that even if somebody pregnant or having her period comes into the house or holds the baby, nothing will happen to the baby, no harm will come.” (FGD 9)

- Age unknown grandmother TBA, Choma FGD participant

However, if the newborn falls ill nonetheless, a respondent recommended protecting the newborn by using a home-based remedy that aims to protect the baby from becoming a fool or stupid.

“If you see the baby is sick, they will get *sinzi* (charcoal), they will burn it and they will mix it with water and blow in the baby's ears on both sides [...] the one who is pregnant or who is attending her period is the one who blows into the baby's ears. This prevents the baby from being a fool, somebody *chiyanga* (a fool, stupid).” (FGD 9)

- Age unknown grandmother TBA, rural Choma FGD participant

### Placenta and Cord Disposal

Many of the disposal practices for the placenta and umbilical cord were driven by a fear of the infant being cursed by another community member wishing harm to the child. It was commonly believed that there were individuals in the community that if able to obtain the placenta, umbilical cord, or detached cord stump, benefited at the expense of the child and the mother. It was reported that these individuals would steal these items and mix it with a variety of herbs for personal gain; however, the exact methods were unknown.

“If it [placenta] is just thrown some people will use it for their own selfish reasons, like use [it] to make the baby unable to have children or [to] put it [placenta] in the medicine.” (IDI 6)

- Age unknown, female CHW, rural Choma FGD participant

“Bad people can take it [placenta] and your baby can die. Some people will use it for their business to prosper.” (FGD 30)

- Age unknown, breastfeeding mother, urban Livingstone FGD participant

With this in mind, a number of practices reported how the disposal of the placenta and the remaining cord was done exercising extra precaution -- keeping its disposal clandestine. Most commonly, the placenta and cord were buried in the ground either inside or behind the house or outside in the bush. A number of respondents preferred to bury the placenta and cord inside the home to prevent young children or scavenging animals such as dogs and pigs from unearthing them as well as to ensure it stays [Table 4]. A few respondents reported cultural beliefs and practices associated with burying the placenta and cord in a certain way to increase future fertility and influence the gender of future children. Respondents believed that uncovering the placenta and cord would result in the child becoming sterile. Alternative methods of disposal included burning and throwing into a pit latrine. 

**Table 4:** Comments Regarding Placenta, Cord, and Cord Stump Disposal.

Theme	Quotes	Respondent Characteristics
Placental and cord disposal	“In the house where the delivery was conducted, they dig a small hole and bury it there […] where nobody will see her.” (IDI 3)	58 year old, male traditional healer, Choma
	“When the cord drops off they will get it and bury it in the house under the bed. [If buried outside] some children sometimes will just pick it and put it in the mouth. So the children will be playing with it, or maybe the dog will come and get it.” (IDI 17)	39 year old, male traditional healer, Mazabuka
	“If it’s the first child, then the placenta, […] goes in the hole. If she wants to have a baby boy next time, she turns the placenta. If she wants another girl, she will leave it like that she wont turn it, than afterwards she covers that hole with soil. Than you, the one who has taken her there, you will bring a big stone and put it right there where you have buried the placenta.” (FGD 18)	Age unknown, untrained TBA, Mazabuka
	“Traditionally they will take a piece of umbilicus, put it with a piece of charcoal and dispose it to the Western side, that is where they believe good fortune is coming from. And so the baby can live longer […] they say the umbilicus should be buried with ashes. If not, then she won't conceive again [...] they should wait until the older person comes, they need an older person to come and bury it.” (FGD 36)	Age unknown, TBA, Livingstone
	“Throw in toilet to avoid witches [...] like my sister baby's cord was buried but a witch took it, so the baby is not with us any more.” (FGD 14)	Age unknown, breastfeeding mother, Mazabuka
Cord stump disposal	“Put it between the roof and the wall so that a rat can take it and eat it.” (FGD 1)	Age unknown, untrained TBA, Choma
	“You sweep in the bedroom where the mother and baby is, you don't throw away the dirt, instead you sweep it to the corner of the room, until the cord falls off that is when you throw that dirt together with the cord” (IDI 12)	58 year old, female untrained TBA, Mazabuka
	“When mothers meet for a gathering, if one baby has a strong medicine, other children who are not protected will get sick. To protect your baby when the cord drops, you tie it (the cord) to the chitenge. You get the cord, wrap it in a small piece of cloth and then sew it to the chitenge which you use to wrap your baby around so that even if you meet someone with medicine your baby will not be sick.” (FGD 22)	Age unknown, breastfeeding mother, Monze

Respondents often reported they would dispose of the cord stump where they had buried the placenta and the rest of the cord [Table 4]. However, there are also a number of other practices described, such as: sweeping the fallen cord to be thrown out with the dirt in the home, or allowing the fallen cord to be eaten by rats, cockroaches or ants. Another practice was to use the fallen cord piece as a type of “preventative medicine” for the baby. 

Culminating the cord detachment process, respondents reported a number of cultural beliefs and practices celebrating the detachment of the cord including the preparation of chicken, *nshima* (traditional maize meal), and a traditional relish. The mother would partake in this meal signifying that her period of home confinement was over.

“They would slaughter a chicken to celebrate *walohya* *mutunbu* (the cord has dropped).” (FGD 19)

- Age unknown female TBA, Monze FGD participant

“They cook the *nshima* so that now, the mother will mix with other friends. She will be free now to be mixing with friends because the cord has dropped. This *nshima* shows that now today the cord has dropped.” (IDI 21)

- 66 year old, female traditional healer, Monze

This meal would be prepared and eaten in a special way by the family members. Respondents reported that all but a small portion of the relish would be consumed. This was believed to confer both a blessing of prosperity and a protection against dullness to the newborn.

"In the past we use to have *Chitumbtumku*. This is a meal of *nshima* and relish that is prepared the following morning after the cord falls off […] the elderly women will eat that meal but leave a bit of each, then the mother to the baby will be requested to sweep the house including the bedroom where the cord was thrown, this then signifies the beginning of her starting her house chores. The left food signifies that when the newborn baby grows up, he will be able to feed many people." (FGD 26)

- Age unknown grandmother, Monze FGD participant

## Discussion

Our study in the Southern Province of Zambia demonstrates the vast diversity of knowledge, disease constructs and practices regarding cord function, tying, cutting, applications, care, and disposal. We found that integration of traditional practices with a Western biomedical model of care was common in Southern Province. This blending of health beliefs and health systems lends itself to the creation of a health systems model that could guide future policy or program design to work synergistically with existing beliefs and practices. In this discussion, we will first discuss and compare cord care practices, cultural beliefs and care seeking patterns across several cultures based on the available evidence and incorporating our own findings and current Zambian Ministry of Health policies. Second, we offer a framework for consideration as an approach to generating more effective health policy and programming.

### Comparison of Cord Care Across Cultures

Several studies have described various aspects of newborn care in a variety of settings [9,11-24]; here, the inclusion of Zambian data creates an opportunity to make comparisons across regions. Study respondents described a variety of tools used to cut the cord. Traditional tools such as bamboo shoots are also commonly used to cut the cord in Bangladesh and India [35-38]. Despite these unconventional tools, new razor blades were the most preferred tool due to affordability, efficiency, and capacity of single use and low risk of blood-borne disease transmission (i.e. HIV/AIDS). However, there was no further sterilization of the tools due to a belief that “new is clean.” Razor blades are readily available from most shops and chemists; typically these razor blades are wrapped in wax paper and exposed to open air and dust, which raises concern for tetanus or other forms of bacteria. Although a study from Sudan found the type of tool used to cut the cord not significantly associated with neonatal tetanus [39], there is ample evidence to support that unsterilized tools may increase the risk of neonatal tetanus or omphalitis [13,37,40,41]. 

WHO recommends delayed cord clamping in order to prevent anemia in newborns [14, 42]. Timing of tying and cutting the cord differed amongst respondents. Although most respondents favored to cut the cord before the placenta was delivered, the fear of retained products influenced others to delay cord cutting until after placental delivery. This fear was documented in Bangladesh as well, where it was believed cutting the cord before the placenta was delivered would cause harm to the woman by the placenta moving into her chest [38]. The rationale for delayed cord clamping in Zambia differs than the rationale behind the current WHO policy for delayed cord clamping. This presents an opportunity for policy makers and program designers to find a synergistic overlap between belief systems that would result in positive behavior change that satisfies multiple stakeholders.

Although dry cord care was widely promoted by the WHO as the standard of practice at the time of our study, we found many who practiced alternative approaches to cord care in this region of Zambia. In many cultures it is strongly believed that the cord should not become dry, so the practice of applying substances to the cord stump aims to make the cord soft, allowing it to separate and heal easily and quickly [43]. Conversely, participants also reported that if the cord was not detaching quickly enough, drying agents might assist in accelerating the process. Although many respondents reported that signs of infection such as umbilical pus and redness would prompt care seeking at the clinic, many others reported the medicinal benefits of applications such as breast milk, charcoal powder, dung and mud. In the biomedical model, application of dung, dust, charcoal, wasp nest powder and other substances is considered a potentially harmful practice, increasing infection risk. Recent publications regarding the effectiveness of 4% chlorhexidine as an umbilical wash to prevent umbilical cord infection from Bangladesh, Nepal, and Pakistan have prompted WHO to consider revising global policy regarding cord care [10,35,44]. Ongoing studies in Zambia and Tanzania will provide efficacy and effectiveness data upon completion for sub-Saharan Africa [45,46]. Understanding the cultural context of cord applications, what prompts an application, who decides when and what application to apply are critical inputs when considering new cord care policy [47]. 

The manner in which the cord drops was of universal concern to our respondents. It was generally believed that faster cord detachment is preferable. This is a particularly important point when considering that currently promoted cord care antiseptics may prolong time to cord separation [48]. Mullany et al. found in Nepal that cord detachment was prolonged with 4% chlorhexidine use as compared to dry cord care (dry cord care mean 4.24 ± 1.6 days; chlorhexidine mean 5.32 ± 2.4 days) [49]. This observation was also made in an interim analysis from the Zambia chlorhexidine application trial (dry cord care mean 4.65 ± 2.25 days; chlorhexidine mean 7.33 ± 3.84 days, p< 0.001) [50]. Prolonged time to cord detachment may ultimately affect chlorhexidine acceptability as an intervention. Respondents associated the behaviors of parents, family and visitors with how fast the cord drops. A father’s infidelity, a mother beginning chores, or even pregnant or menstrual women visiting the baby before the cord drops were perceived to prolong the process and thus should be avoided. When designing public health interventions, one must pay particular attention to the concerns that visitors raise for newborn health. Many proposed newborn health interventions involve home visitation by health workers; this concept needs to be vetted by communities where visitation of certain people (e.g pregnant women, menstruating women) are prohibited. When the cord does drop, respondents perceived it falling on the pubic area to lead to infertility. These findings are consistent with another study in Zambia by Maimbolwa et al., where participants expressed concern that sexual acts of the parents, including infidelity, could affect future fertility of the mother and the infant [25]. 

There was considerable concern about how the placenta and umbilical cord should be disposed. Many respondents expressed a belief that improper disposal of the placenta (e.g. through inadequate burial) could lead to a curse on the baby if found by someone in the community who wished the baby misfortune or harm. Therefore, extra precautions regarding the placenta should be taken. A similar study of traditional care practices in India and Tanzania also reported that the placenta should be buried to safeguard the child from evil spirits [16,51].

### A Proposed Framework

**Figure 4:**
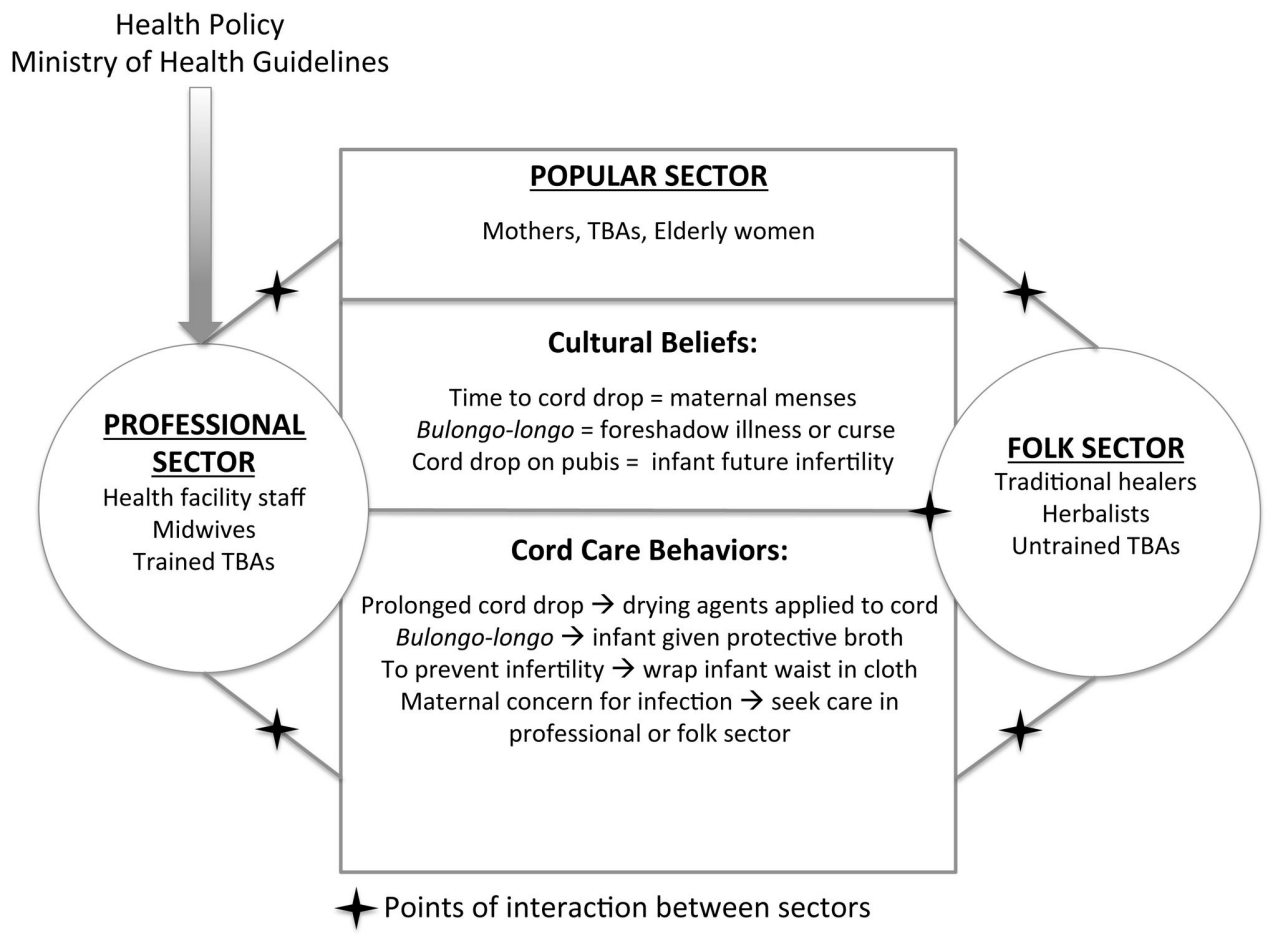
Adaption of Kleinman’s health care system as a cultural system framework with illustrative examples of cord beliefs in Southern Province, Zambia.

Kleinman’s framework of explanatory models of disease and illness enables the comparison of health care systems in any cultural context. Kleinman proposes that the healthcare system is a cultural system in and of itself with three sectors: popular (shaped by individual, family and community beliefs); the folk sector (represented by non-professional healing specialists); and professional (western medical professional or professionalized indigenous healing traditions) [52]. Applying this framework to our findings, we found that the main stakeholders who shape the popular sector are the newborn’s mother and older women in the community, specifically grandmothers, mother-in-laws, and traditional birth attendants. The folk sector is comprised of traditional healers and untrained traditional birth attendants. Lastly, trained TBAs, midwives and health facility personnel define the professional sector. It is debatable whether to include the trained TBAs in the professional sector or the folk sector as their formal training ceased 10 years prior. We decided to place trained TBAs in the professional sector because often (although not uniformly) they perform deliveries and other health promotion tasks at health facilities and may have a greater volume of deliveries and experience than untrained TBAs. Perhaps most importantly, community members view them as an extension of the professional healthcare system regardless of whether their skillset is different from untrained TBAs. Below we have adapted Kleinman’s model for Southern Province, Zambia using a few illustrative examples of cultural beliefs regarding cord health and illness and their related cord care behaviors [Figure 4]. The stars indicate areas where the popular, professional and folk sectors interface, presenting potential opportunities for behavior change interventions to reduce infection risk. Note that the only place where policy and clinical guidelines currently have input is the professional health sector. If we allow ourselves to view the healthcare system as a cultural system influenced by all sectors and the beliefs and behaviors practiced within, policy and programming can be designed in collaboration with each sector, allowing for greater uptake and sustainability of mutually acceptable practices. 

### Strengths and Limitations

One of the strengths of this study is the large sample size and diversity of participants. There was representation from a variety of perspectives on newborn care including recent mothers, long practicing TBAs and midwives, and Ministry of Health employees at the district and community levels. All IDIs and FGDs were conducted in the native language of the respondent in hopes of maximizing respondent’s comfort and ease of sharing. One limitation of the study is that many respondents shared practices or beliefs that they “had heard” others practiced. What is unclear is how many of the primary respondents personally hold these beliefs or actually followed these practices. Because it was not a random sample, we cannot comment on frequency of cord application or commonality of beliefs expressed. 

## Conclusions

As global pressure increases to reach Millennium Development Goal 4, to reduce child mortality in half by 2015 from the 1990 level, increased attention will be given to the earliest minutes, hours, and days of life. As research efforts and interventions further explore cord care practices, it is important to understand the context, existing beliefs and practices of each region where cord care policy may change. The findings of this study present a diverse and rich traditional approach to caring for newborns and the cord. In addition to sharing our findings regarding cord care in Southern Province, Zambia, we offer a framework to use by any health care system that is considering policy change. This framework will assist policy makers and program implementers to identify all sectors that comprise the health care system and identify opportunities for effective change and collaboration. With awareness of existing cord care practices, we will be better able to build community-based interventions that are specific to the cultural beliefs, traditions, and practices of the region, and ultimately reduce the neonatal mortality burden.

## Supporting Information

Table S1 **Glossary of Chitonga Words or Phrases Used in FGDs or IDIs.**
(DOCX)
