# Supplementary material for: Local Perceptions, Cultural Beliefs and Practices That Shape Umbilical Cord Care: A Qualitative Study in Southern Province, Zambia
Source: PLoS One. 2013 Nov 7;8(11):e79191. doi: 10.1371/journal.pone.0079191 (PMC3820671; doi:10.1371/journal.pone.0079191)
Supplement: Table S1 — Glossary of Chitonga Words or Phrases Used in FGDs or IDIs. (DOCX) [file pone.0079191.s001.docx]

**Supporting Information**

**Table 1. Glossary of Chitonga Words or Phrases Used in FGDs or IDIs**

| Chitonga | Meaning in English |
| --- | --- |
| chifufuti | abdominal pain or cramps similar to those experienced during menses |
| chitenge | traditional printed fabric worn by women as a wrap |
| chitumbtumku | celebratory meal |
| chiyanga | one who is considered a fool or stupid |
| dezhya | stalk peelings from maize cob |
| loma | wasp nest |
| loozi | fiber from the bark of a tree |
| luhumwe | distended abdomen |
| lunyoolo | stones or metals |
| mabono | wild fruit from which oil can be expressed |
| masoto | respiratory illness or bad air |
| mukunku | type of tree |
| nshima | traditional maize meal |
| sinzi | charcoal |
| walohya mutunbu | the cord has dropped |
